# It is time anti-CGRP monoclonal antibodies be considered first-line prophylaxis for migraine

**DOI:** 10.1590/0004-282X-ANP-2022-S112

**Published:** 2022-08-12

**Authors:** Gabriel Taricani Kubota

**Affiliations:** 1 Universidade de São Paulo, Faculdade de Medicina, Hospital das Clínicas, Departamento de Neurologia, São Paulo SP, Brazil.; 2 Instituto do Câncer do Estado de São Paulo, São Paulo SP, Brazil.

**Keywords:** Migraine Disorders, Antibodies, Monoclonal, Calcitonin Gene-Related Peptide Receptor Antagonists, Cost-Benefit Analysis, Transtornos de Enxaqueca, Anticorpos Monoclonais, Antagonistas do Receptor do Peptídeo Relacionado ao Gene de Calcitonina, Análise Custo-Benefício

## Abstract

The result of more than thirty years of research, anti-CGRP monoclonal antibodies are currently the state of the art for migraine preventive therapy. Their efficacy and safety, supported by an already large and growing body of evidence, are added by many other advantages: an early onset of action, favorable posology, negligible pharmacological interaction, and a broad-reaching efficacy in many challenging clinical contexts. When compared to standard prophylactics, these novel medications seem at least as efficacious, clearly more tolerable and, consequently, with a superior adherence profile. Furthermore, recently published analyses indicate that they are cost-effective, especially among those with chronic migraine. Yet, current guidelines endorse their use only after multiple other preventives have failed or have been deemed not tolerable. Although this recommendation may have been sensible at first, the now available data strongly point that time has come for anti-CGRP monoclonal antibodies to be acknowledged as first-line treatments for migraine patients with severe disability. For these individuals, delaying treatment until several other alternatives have failed incurs in significant losses, both economically and to many relevant aspects of their lives.

## RESUMO

Frutos de mais de 30 anos de pesquisa, os anticorpos monoclonais anti-CGRP são atualmente o que há de mais moderno no tratamento preventivo da migrânea. À sua eficácia e segurança, já bem estabelecidos por um grande corpo de evidências, acrescentam-se outras vantagens: um início precoce de ação, posologia favorável, mínima interação farmacológica, e eficácia comprovada em uma variedade de contextos clínicos frequentemente desafiadores. Quando comparados a outros profiláticos, estas medicações aparentam ser ao menos tão eficazes, evidentemente mais toleráveis e, portanto, com melhor perfil de adesão. Ademais, estudos recentemente publicados indicam que elas são custo-efetivas, especialmente entre pacientes com migrânea crônica. Ainda assim, as diretrizes atuais orientam o seu uso apenas caso haja refratariedade ou intolerância a múltiplos outros preventivos. Apesar de esta recomendação poder ter sido sensata *a priori*, os dados disponíveis atualmente corroboram que já é tempo de estes anticorpos monoclonais serem reconhecidos como tratamentos de primeira linha para a migrânea associada à incapacidade grave. Para estes pacientes, demorar a oferecer este tratamento até que outras múltiplas alternativas tenham falhado, leva a perdas significativas, tanto economicamente quanto em múltiplos outros aspectos relevantes das suas vidas.

## INTRODUCTION

Since the role of calcitonin gene-related peptide (CGRP) in migraine pathophysiology was first proposed in 1985, research in this field have come a long way, culminating in the publishing of the encouraging results of anti-CGRP monoclonal antibodies (anti-CGRP mAb) phase 3 trials in 2017, and the introduction of these medications in the American and European markets in the following years^1^ ^,^ ^2^. It should be highlighted that this was not only a major milestone in the clinical management of migraine but, from a historical standpoint, it was a revolution in the framework of migraine therapeutic development^3^. Indeed, until then, most advances in pharmacological prophylaxis for this condition had resulted from sheer serendipity, i.e., from repurposing drugs *a priori* developed for other diseases, such as hypertension, epilepsy or mood disorders, for migraine treatment, largely based on clinical empiricism^4^. Conversely, by targeting a specific molecule closely linked with its pathophysiology, anti-CGRP mAbs marked the dawn of the precision medicine era for migraine prophylaxis.

 The clinical significance of the “target-based” properties of these medications can be observed in data stemming from the many clinical trials and prospective cohorts published since. While retaining an efficacy which is at least similar to that of other well-established drug prophylaxis, they have shown to deliver a much-improved tolerability and safety profile^5^ ^,^ ^6^. That is not to mention a streak of very significant other advantages, including an early onset effect; favorable posology^7^ ^-^ ^9^; negligible pharmacological interaction^10^; and a wide-range efficacy in many challenging clinical contexts such as medication-overuse headache (MOH), menstrually-related and multidrug resistant migraine^11^ ^-^ ^14^.

 However, in spite of all these advantages, recent guidelines on this topic seem to be less than encouraging when it comes to incorporating anti-GCRP mAbs into daily clinical practice. In fact, currently, both the American Headache Society and the European Headache Federation recommend the introduction of these medications only after at least 2 other well-established prophylaxis have failed or have been found to be not tolerable^15^ ^,^ ^16^. While this caution may have been well justified at first, would the mounting favorable evidence and clinical experience gathered from the past 5 years not suffice to recommend their use as first-line treatment now?

## WHAT IS EXPECTED FROM A FIRST-LINE THERAPY?

 Firstly, it is important to acknowledge that making recommendations on medical treatments is no simple task. At first glance, the core concept behind any recommendation seems quite straightforward: deciding on the balance between the desirable and undesirable effects of a given intervention^17^. Certainly, the quantity and quality of the available body of evidence regarding their efficacy and safety plays an important role in this decision. In this sense, standardized methods for the assessment of evidence, such as the GRADE framework, have been developed and are widely used^18^. 

 However, if quality of evidence (QoE) was all it took, the sheer number of well-designed large, multicenter, double-blind clinical trials pointing to the significant efficacy and tolerability of anti-CGRP mAbs would render this discussion pointless. QoE, in fact, only reflects the certainty to which the available data regarding an intervention is consistent and generalizable. This is indeed an important aspect of a recommendation, but not the only one, and frequently not even the most important^17^. 

 Indeed, the decision on whether a certain treatment should be considered first-line for a given disease must also encompass: the nature of the benefits and risks of this treatment, and their significance for the main stakeholders; the expected size of its beneficial effects; the cost-effectiveness; the potential impact on reducing health inequities; how it fares when compared to other available therapies; and its feasibility and acceptability^17^. Noticeably, current evidence supports that anti-CGRP mAbs fulfill all of the above criteria.

## WHAT BENEFITS ARE EXPECTED FROM ANTI-CGRP MABS?

**Figure 1.**
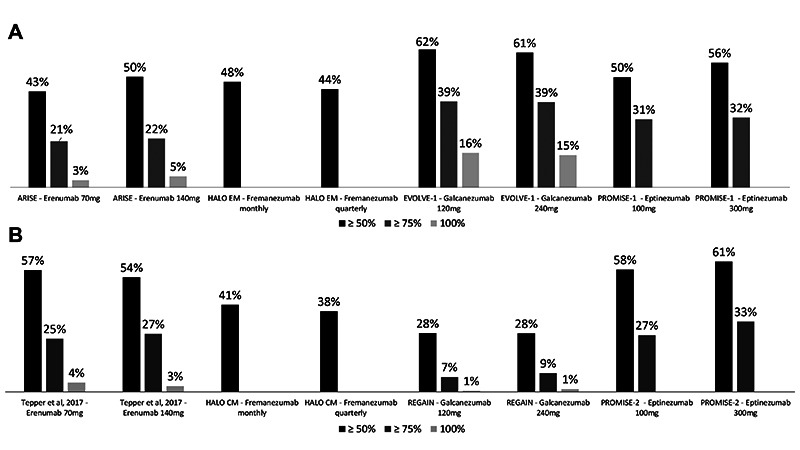
Results for the 50%, 75% and 100% response rates of the pivotal trials of anti-CGRP monoclonal antibodies for migraine treatment. A: episodic migraine; B: chronic migraine.

 All pivotal placebo-controlled trials of erenumab (STRIVE, ARISE and Tepper et al., 2017) ^19^ ^-^ ^21^, fremanezumab (HALO EM and HALO CM)^22^ ^,^ ^23^, galcanezumab (EVOLVE-1&2 and REGAIN)^24^ ^-^ ^26^ and eptinezumab (PROMISE 1&2)^27^ ^,^ ^28^ reached their primary endpoint of reducing monthly migraine days (MMD) for both episodic (EM) and chronic migraine (CM). This indicates an overall class benefit due to their shared mechanism of action in blocking CGRP pathway. Criticism may arise from the apparent small effect size obtained for this endpoint, which ranged from 1.3 to 4.6 and from 1.7 to 2.6 MMD for EM and CM, respectively. However, it should be noticed that these values fall in line with those observed in most previous trials for well-established first-line migraine therapies, i.e., onabotulinumtoxin A, topiramate, valproate and candesartan. Furthermore, 50% response rates (i.e. proportion of subjects who achieved ≥ 50% reduction in MMD frequency), a more palpable measure of the clinical utility of a prophylactic intervention, ranged approximately from 40 to 60% and from 30 to 60% for EM and CM, respectively (Figure 1).

This chart summarizes the available data for the response rates found in the pivotal randomized clinical trials of erenumab (STRIVE, ARISE and Tepper et al, 2017) ^19^ ^-^ ^21^, fremanezumab (HALO EM and HALO CM)^22^ ^,^ ^23^, galcanezumab (EVOLVE-1&2 and REGAIN) (24-26) and eptinezumab (PROMISE 1&2)^27^ ^,^ ^28^.

 Besides reducing migraine frequency, anti-CGRP mAbs produced relevant benefits in a series of patient-reported outcome measures. For example, among EM and CM subjects who completed the 52-week HALO fremanezumab extension study, large proportions reported improved anxiety (67.9%) and depression (64.7%) levels, better sleep quality (56.7%), better work/school performance (85.4%), better quality of time spent with family/friends (83%) and more enjoyment from leisure activities (81%)^29^. Improvements in functionality and quality of life measures have also been reported in erenumab and galcanezumab trials^30^ ^,^ ^31^.

 Furthermore, open-label studies have demonstrated that these benefits are maintained in the long run. A 5-year prospective cohort with 383 EM patients treated with erenumab observed that reductions in MMD were retained throughout the follow-up, as well as the improvements in disability, headache impact and migraine-specific quality of life measures^32^. Of notice, this study found a 50% response rate in 71% at the 5-year follow-up, and that 35.5% had complete remission of their MMD^32^. These findings were also supported by those of other 12-month open-label cohorts with galcanezumab^33^ ^,^ ^34^ and a 12-month randomized trial with fremanezumab^35^.

## CAN ANTI-CGRP MABS REDUCE INEQUITIES IN MIGRAINE MANAGEMENT?

 Another very positive aspect of this medication class is its ability to deliver the above mentioned benefits to groups of subjects who typically fare poorly with current standard-of-care drug prophylaxis. For example, among the subset of migraineurs with medication-overuse headache (MOH), post-hoc analysis of the pivotal trials for erenumab^36^, galcazenumab^37^, fremanzeumab^38^ and eptinezumab^39^ showed that their efficacy in reducing MMD was retained, and remained similar to that observed for non-MOH patients. Moreover, in a prospective 6-month real-life cohort including 139 CM patients (71.2% with MOH), treatment with erenumab or galcanezumab resulted in similar 50% response rates for MMD reduction both in MOH (63.6%) and non-MOH (57.5%; p=0.50) subjects^11^. Very interestingly, although 60.6% of the MOH patients ceased to fulfill criteria for this condition at the end of this study, no detoxication protocol nor education to stop acute medication were administered prior to anti-CGRP mAb treatment^11^. Also, in another post-hoc analysis, the subset of EM patients with menstrually-related migraine enrolled in STRIVE was shown to benefit similarly to those without this condition from erenumab treatment^12^.

 Anti-CGRP mAb treatment also may herald hope for those who were refractory to multiple well-established prophylaxis, or are unable to tolerate them. This specific subset of patients has been directly examined in phase 3b clinical trials for erenumab (LIBERTY)^13^, galcanezumab (CONQUER)^40^, fremanezumab (FOCUS)^41^ and eptinezumab (DELIVERY)^42^. These studies included EM and/or CM subjects who had failed or were deemed unable to tolerate 2 to 4 standard drug prophylaxis. As with the pivotal trials for these medications, all of them reached their primary endpoints of reduction in MMD. What is more, the achieved magnitude of effect for 50% response rate ranged approximately from 30% to 50%. These are very promising figures for such a challenging group of patients, especially when considered the lower than usual placebo effect found in these trials. 

 Additionally, anti-CGRP mAbs may also benefit subjects who have failed the only available first-line parenteral migraine prophylaxis: onabotulinumtoxin A. A real-life prospective cohort including 150 subjects who had partial or no significant response to onabotulinumtoxin A, found that a 3-month treatment with galcanezumab or erenumab resulted in a 50% response rate of around 50% in MMD reduction^43^. Results from a post-hoc analysis of the pivotal galcanezumab trials also supported these findings^44^.

## WHAT ARE THE OTHER ADVANTAGES OF ANTI-CGRP MABS?

**Figure 2.**
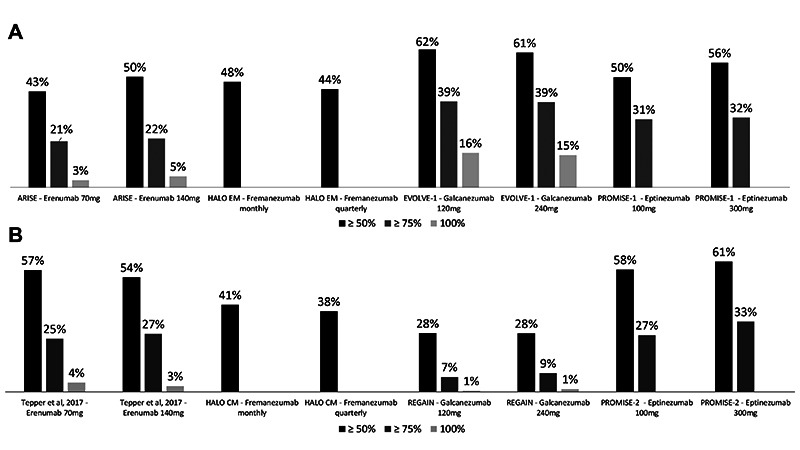
Discontinuation rates in randomized clinical trials of pharmacological migraine prophylactic treatments.

 Besides the many already mentioned benefits that these medications bring to the table, they also offer other very relevant advantages. The most important one, perhaps, is its favorable posology and adherence profile. Indeed, oral migraine prophylaxis is frequently hindered by high drop-out rates, even at medium-term follow-up. A systematic review of observational and clinical trials with propranolol, amitriptyline and topiramate found that adherence rates varied from 21 to 80% at 6 months, and from only 35 to 56% at 1 year^45^. Adverse events were the most common cause for discontinuation^45^. This major drawback for migraine therapy was initially tackled by the introduction of the parenteral treatment with onabotulinumtoxin A for CM, administered quarterly. Randomized trials with this medication found drop-out rates as low as 2 to 3%^5^. Likewise studies with anti-CGRP mAbs, administered parenterally monthly or quarterly, observed very low discontinuation rates ranging from 0 to 4% (Figure 2).

Data from Vandervorst *et al*, 2021 (5).

 Another advantage is the rapid onset with which anti-CGRP mAbs provide their effect. While conventional oral drug prophylaxis require weeks to deliver improvements in migraine, mounting evidence indicates that significant benefits can be observed as early as in the first day of therapy for galcanezumab^7^, and in the first week for erenumab^9^ and fremanezumab^8^ ^,^ ^46^. Of special interest, besides its established efficacy in reducing MMD, eptinezumab has also been shown to be useful in treating acute migraine attacks. In a recently published randomized trial, it resulted in significantly superior headache pain freedom and resolution of the most bothersome symptom rates as early as 2 hours after infusion, when compared to placebo^47^.

Finally, anti-CGRP mAbs have a very favorable pharmacokinetic profile. Instead of being metabolized by liver enzymes, these medications are cleared by general proteolytic degradation pathways^10^. This reduces significantly the risk for clinically significant pharmacological interaction with other drugs, such as antiepileptics, anticoagulants and hormonal contraceptives. 

## DO WE HAVE ENOUGH DATA ABOUT ANTI-CGRP MABS SAFETY?

 Long before the publishing of the results of the first phase 3 trials of anti-CGRP mAbs, reasonable concerns were raised regarding the risks of interfering in the CGRP pathway. This is because CGRP has been shown to play significant roles in many physiologic processes, especially: inflammation, wound healing, vasodilatation and insulin release and resistance^48^. However, it must be highlighted that this molecule is not the only one implied in these processes, nor are anti-CGRP mAbs able to completely block its pathway. In fact, rimegepant (a small molecule CGRP receptor antagonist) has been observed to be effective in treating acute migraine attacks even in the setting of erenumab treatment^49^, indicating that a significant CGRP pathway activity persists in spite of the use of this mAb. 

 Despite these concerns, the only adverse reaction that has been consistently found to be statistically more frequent with anti-CGRP mAbs in the high-quality placebo-controlled trials that have since been published are of local nature, i.e. injection-site pain, erythema and pruritus^50^. It is important to highlight that these adverse reactions were generally mild-to-moderate in severity, did not increase with the number of doses received, and very rarely led to treatment discontinuation^51^. An exception is made for erenumab trials, which also reported a higher frequency of constipation when the 140 mg dose was administered. However, this collateral effect was also described to have been mild to moderate and easily managed^52^. Moreover, its frequency reduced significantly throughout the long-term open-label extension phases of these studies^52^. It should be highlighted that erenumab safety has been assessed in a 5-year open-label extension study that enrolled subjects who had participated in a phase 2 randomized trial with this medication for EM^32^ ^,^ ^53^. This was one of the longest cohorts to have ever been published among migraine prophylactic drugs, and it found no new safety signals, nor increased rates of adverse effects, in relation to the double-blind trial phase^32^. Furthermore, pooled results from 2 small double-blind placebo-controlled trials with eptinezumab showed that this medication was well tolerated and not associated with metabolic effects among overweight/obese patients, nor those who suffered from type 1 diabetes^54^. 

 Post-marketing surveillance studies have also portrayed an overall reassuring picture so far. It must be acknowledged that the FDA Adverse Event Reporting System (FAERS) has identified post-marketing cases of possible association between erenumab and new-onset hypertension^55^. This has led FDA to add a warning statement in the prescription label of erenumab in the United States. However, in the more than 1,300 migraine patients that were treated with erenumab in phase 2 and 3 randomized clinical trials, this adverse event has not been identified, nor any other significant cardiovascular collateral effect could have been attributed to this medication^56^. What is more, when data from clinical trials and post-marketing surveillance studies are pooled together, the exposure-adjusted incidence of hypertension was as low as 0.144 per 100 patient-years^57^.

## DO ANTI-CGRP MABS WORK BETTER THAN OTHER MIGRAINE THERAPIES?

 Although this is a very relevant topic, as in many other fields of Neurology, there is a lack of direct data from head-to-head trials that may help address it. However, so far available data supports a clear-cut advantage of anti-CGRP mAbs over standard first and second-line therapies. 

 To the best of my knowledge, the first and so far the only head-to-head trial to have compared an anti-CGRP mAb with a first-line migraine prophylaxis was HER-MES^58^. This was a phase 4 double-blind randomized trial, with a double-dummy design, which compared topiramate (50-100mg/day) with erenumab (70-140mg/monthly), during a 6-month follow-up period. Its primary endpoint was the rate of medication discontinuation due to an adverse event, but 50% response rate was also examined. This trial enrolled 867 subjects, most of whom naïve to prior prophylactic treatment (59.8%) and with EM (88.9%)^58^. Drop-out rates due to adverse events were significantly lower in the erenumab group (10.6%), compared to the topiramate one (38.9%; OR 0.19; p<0.001). Also, in a modified intention-to-treat analysis, the 50% response rate for MMD reduction was significantly larger for erenumab (55.4%) than topiramate (31.2%; OR 2.76; p<0.001)^58^. Although discontinuation rates for erenumab were indeed larger than those reported in previous trials, as the authors have pointed out, this may have been justified by a nocebo effect resulting from the double-dummy design^58^. Of note, topiramate is currently considered one of the most efficacious prophylactics, and the oral one with highest level of evidence for CM treatment^59^ ^,^ ^60^. 

 Besides HER-MES, comparison with other standard preventive medications has been made through indirect comparison studies^5^ ^,^ ^6^ ^,^ ^61^. One of the most broad-reaching of these, by Vandevorst *et al*, included phase 2 and 3 trials of all 4 available anti-CGRP mAbs, and of currently well-established prophylactics (including topiramate, valproate, beta-blockers, candesartan and onabotulinumtoxin A)^5^. It showed that, as a class, anti-CGRP mAbs appear to be clearly superior in terms of tolerability, and at least as efficacious (and possibly more so) than other first-line treatments^5^. QoE was also generally higher for this novel medication class than for the others^5^. 

 Finally, anti-CGRP mAbs also have advantages over onabotulinumtoxin A, a parenteral alternative with similar cost and widely considered first-line therapy for CM^16^ ^,^ ^59^. On one hand, indirect comparison studies between pooled randomized trials of these medications have suggested similar efficaciousness for CM^5^ ^,^ ^61^. On the other, as aforementioned, some studies have demonstrated that anti-CGRP mAb treatment may reduce significantly MMD among subjects who had previously fared poorly with onabotulinumtoxin A^43^ ^,^ ^44^. Onabotulinumtoxin A has not been shown to provide consistent benefits for EM, differently than anti-CGRP mAbs^62^. Additionally, controversy still remains about the efficacy of onabotulinumtoxin A in treating CM associated with MOH^63^. 

Moreover, there are some other practical advantages to this parenteral counterpart that should be mentioned. Firstly, anti-CGRP mAbs administration, made though a single injection, is less discomfortable than the 31 to 39-injection PREEMPT protocol. Moreover, onabotulinumtoxin A is applied in the cephalic and neck segments, including frequently tender regions and areas in which migraine-induced scalp allodynia occurs. Conversely, erenumab, fremanezumab and galcanezumab are administered in distant sites, subcutaneously. Additionally, performing the PREEMPT protocol requires a specifically trained healthcare provider. Accessibility to such professionals may be limited in some locations, or under certain contexts. One recent example was the COVID-19 pandemic, during which lockdown and other sanitary measures led to the interruption of the activities of many headache clinics where onabotulinumtoxin A was administered; and discouraged many to come to the overwhelmed healthcare services to receive it. This resulted in significant delays in the treatment with this medication, and increased headache frequency^64^ ^,^ ^65^. Contrastingly, aside from eptinezumab (which is administered by intravenous infusion), anti-CGRP mAbs are self-administered with ease, and require very little training.

## A MATTER OF BURDEN

At this point, there should probably remain very few strong arguments against offering anti-CGRP mAbs as first-line treatment for migraine. Except for a key aspect: price. And indeed, while the cost of these novel medications may vary across different locations, it is several times more expensive than any other oral prophylactics. Their prices, however, generally fall in line with those of onabotulinumtoxin A. Given that roughly 14% of the worldwide population suffers with migraine^66^, one could argue it would not be viable to promptly offer it as a first line treatment to all. 

While that might seem sensible at first glance, it is also a gross simplification of the problem. Indeed, when deciding on a treatment, one should not only consider its cost, but also the burden imposed by the disease, as well as the effectiveness of the treatment in reducing it. And, though frequently neglected, the burden of migraine is not a small one. Primary headaches are currently the second major cause of disease-related disability worldwide, largely due to migraine^67^. Migraine in itself is the single most important cause of disability among all neurologic diseases, the sheer number of years lived with disability it results in being larger than that produced by stroke, dementia, Parkinson’s disease and multiple sclerosis summed together^67^. This, of course, comes with a price.

In 2014 it was estimated that migraineurs had an incremental US$ 8,924.00 cost to health insurance services annually, when compared to matched controls in the United States (US)^68^. Most of this resulted from direct costs, including outpatient pharmacy, physician visits, brain imaging and hospitalizations^68^. In Italy, direct annual expenditures due to migraine were estimated in € 1,482.00 *per capita*. The distribution of these costs, evidently, is not homogeneous. Annual direct cost of CM is estimated to be 4.8-fold higher than for EM^68^. Furthermore, among MOH patients, two thirds of whom have migraine^69^, annual direct and indirect *per capita* costs soar up to € 10,533.00 in Italy^70^.

Besides health-related costs, migraine severely hinders work productivity. On average, CM patients lose 4.6 work hours per week due to headache^71^. Collectively, due to presenteeism and absenteeism, migraine results annually in losses amounting to US$ 21.3 billion in Japan^72^, £ 8.8 billion in U.K. and € 122 billion in Germany^73^. In Brazil, the economic burden due to headache-related presenteeism and absenteeism (mostly migraine), was recently estimated in R$ 67.6 billion annually^74^. Individually, it also exacts a sizable cost to the career and professional life of some. CM patients were found to be 19% less likely to be working for pay, when compared to low-frequency EM ones^71^. In the CaMEO study, a landmark prospective longitudinal cohort which assessed migraine epidemiology and burden among 13,064 subjects, around two thirds of CM patients reported that this disease had interfered in their careers^75^. In this research, among CM subjects: 15.2% admitted to feeling a burden to coworkers, 14% to having chosen less demanding jobs, 10.8% claimed to earn less/have missed a raise, and 9.8% felt that their career advancement had been limited due to migraine^75^. Unfortunately, in *My Migraine Voice*, another observational study with 11,266 migraineurs, 27% reported lack of understanding among work colleagues about their condition^76^.

Migraine also very frequently results in losses to some things which cannot really be put a price on. For example, in the aforementioned *My Migraine Voice* study, 64% of respondents reported that the disease had undermined their private life, including: missing on important events such as birthdays and weddings (52%); effects on sex life (49%); avoiding making commitments (50%) and feeling guilty about the impact migraine has on their family (44%)^76^. Moreover, 59% claimed not being able to participate in hobbies/activities they used to and 34% informed having been stopped from engaging in sports or exercise^76^. This burdensome condition also takes a heavy toll on loved ones. In CaMEO study, spouses to CM patients reported reduced enjoyment of time spent with their partner (76.5%); feeling that the migraineur had significantly reduced involvement in family activities (62.3%); resenting having to do everything when the migraineur has a headache (23.4%); worrying about covering the household expenses (33.8%) and about having long-term financial security for their family (40.4%); and that they believed the migraineur would be a better parent if they did not have headaches (43.9%)^77^. In the same study, adolescents to parents who suffered from CM had significantly higher levels of depression (p=.08) and anxiety symptoms (p=.01), compared to those whose parents suffered from EM^78^. They were also more prone to feel they would get along better with their parents of they did not have headaches (43.5% vs. 21.5%, p<.001) and that their migraineur parent had let them down (26.8% vs. 13.4%, p<.001)^78^.

In face of the overwhelming and wide-ranging burden migraine causes, it would not be surprising if cost-effectiveness analysis supported the use of anti-CGRP mAbs. And, in fact, they do. A recently published study weighted the overall cost of systematically prescribing erenumab to the whole indicated German population with the reduction in migraine days and corresponding productivity losses. It found that this would result in savings due to avoided productivity losses amounting to € 26.6 billion, at the incremental healthcare costs of only € 8.4 billion^73^. In line with these results, another study with the US population observed that, even when only direct disease-related costs are considered, erenumab may be cost-effective for CM patients^79^. In Switzerland, cost-effectiveness for this medication was also demonstrated for patients who had failed other prophylactics previously^80^. 

## THE PRICE OF THE DELAY

 All things considered, should anti-CGRP mAbs be acknowledged as a first-line therapy for migraine prevention? Modern individually-tailored clinical practice fortunately leaves little space to broad generalizations such as this. Indeed, although these medications are effective, tolerable and safe, their cost may limit their use for mild low-frequency EM. However, that is not the case for burdensome high-frequency EM and CM patients, especially those who suffer from MOH. For these individuals, delaying treatment until several other alternatives with much slower onset of action, less tolerability, possibly less effectiveness and which are much less prone to be adhered to in the long run, have failed, as *per* current guidelines, has a price. A steep price that will be exacted from both the society’s and the patient’s pockets, as well as from priceless things: quality of life, functionality, relationship with loved ones and dignity.
